# The relationship between hemodialysis patients’ treatment adherence, procrastination, and difficulty in emotion regulation: A cross-sectional study in southeast Iran

**DOI:** 10.3389/fpsyg.2022.1041912

**Published:** 2023-01-16

**Authors:** Fateme Dahaghin Bazrafshan, Zahra Darvizeh, Shokoh Sadat Banijamali

**Affiliations:** Faculty of Education and Psychology, Alzahra University, Tehran, Iran

**Keywords:** treatment adherence, procrastination, difficulty in emotion regulation, hemodialysis, cross-sectional study

## Abstract

**Background:**

End-stage renal disease is a life-threatening condition in which patients require dialysis or kidney transplant. These patients must adhere to the treatment regimen because treatment non-adherence affects their quality of life and health. We conducted this study to predict hemodialysis patients’ treatment adherence based on procrastination and difficulty in emotion regulation.

**Materials and methods:**

We conducted this descriptive correlational study on 218 hemodialysis patients with chronic kidney disease. We used purposive sampling to select participants from six dialysis centers in Kerman, Sirjan, and Rafsanjan. The measuring tools included the end-stage renal disease adherence questionnaire, general procrastination scale, decisional procrastination scale, and difficulty in emotion regulation scale. We used the correlation coefficient, regression analysis, and SPSS18 to analyze data.

**Results:**

Our study indicated that among the dimensions of treatment adherence, medication use had a significant, weak, and inverse relationship with general and decisional procrastination. We also found a significant, weak, and inverse relationship between attendance and general procrastination (*p* < 0.05 and *p* < 0.01). But there is no significant relationship between treatment adherence, general procrastination, and decisional procrastination (*p* > 0.05). Multivariate regression analysis revealed a relationship between age, the cause of kidney failure, and treatment adherence (*p* = 0.01 and *p* = 0.02).

**Conclusion:**

Treatment non-adherence causes problems and complications in hemodialysis Patients, and disrupts their course of treatment. Therefore, it is necessary to identify the factors influencing non-adherence of patients undergoing hemodialysis and improve their treatment adherence, and thus their quality of life.

## Introduction

Chronic kidney disease is a non-communicable disease with significant mortality risks worldwide (GBD 2016 Disease and Injury Incidence and Prevalence Collaborators, 2017). The incidence and prevalence of this disease in the world and in Iran is increasing. The incidence of this disease is different in different regions of the world. However; in general, the incidence of this disease in most countries is more than 200 cases per million people per year. The prevalence of ESRD in the United States, Europe and Iran is estimated at 1,500, 800, and 360 cases per million (Ahmadi et al., 2022). End-stage renal disease is a life-threatening condition in which patients require renal replacement therapy, such as dialysis or kidney transplant. Hemodialysis is the most widely used type of dialysis in many countries around the world (Mukakarangwa et al., 2018). Dialysis prolongs survival and improves quality of life in patients with chronic kidney disease (Wang et al., 2017). Compliance with medical regimens, such as hemodialysis is very important to achieve optimal treatment. Hemodialysis patients must both adhere to their treatment regimen and change their lifestyles (Wang et al., 2017). However, hemodialysis patients require adaptation across multiple dimensions. Non-adherence to the prescribed regimen in hemodialysis is a major concern that affects patient outcomes, including survival (Ok and Kutlu, 2020). We considered four dimensions when assessing adherence to the hemodialysis, including attendance, fluid intake restrictions, dietary guidelines, and medication use (Tayebi et al., 2019).

Non-adherence to fluid intake restrictions may cause hypertension, pulmonary edema, congestive heart failure, muscle cramps, nausea, vomiting, anxiety, panic, and low blood pressure during hemodialysis, with an increased risk of hospital stay (Vaiciuniene et al., 2012). Hemodialysis patients often have dietary problems that associate with complications and mortality. Dietary guidelines for hemodialysis patients include restrictions of sodium (Na), potassium (K), protein, and phosphorus (P) (Magnard et al., 2013). Hemodialysis patients must adhere to regular use of medications. Cardiovascular disease is the main cause of high mortality in hemodialysis patients. To avoid secondary risk factors for cardiovascular diseases, including hyperparathyroidism and HT, patients must take 6–12 tablets per day (Ok and Kutlu, 2020). Patients’ medication non-adherence causes health problems and has negative effects on their quality of life. Treatment adherence associates with better health-related quality of life in hemodialysis patients (García-Llana et al., 2013). Many studies addressed factors affecting treatment adherence to improve the clinical results of these patients and overcome existing obstacles. According to researchers, poor economic status, aging, maleness, years of suffering from chronic kidney disease, history of hospital stay due to treatment non-adherence, history of diabetes, history of kidney transplant, side effects and tastes of drugs, and the need to change behavior were predictors of treatment non-adherence (Green et al., 2013; Griva et al., 2013; Mellon et al., 2013; Hadian et al., 2016; Mukakarangwa et al., 2018; Hashemi et al., 2020; Asadizaker et al., 2022).

The theories of self-regulation and social cognition play important roles in treatment adherence interventions. Procrastination is a failure in self-regulation (Ramzi and Saed, 2019) and represents a delayed choice and decision that persists over time, despite numerous opportunities to execute changes. Not all delays are the result of procrastination. In other words, procrastination is the act of unnecessarily postponing decisions or actions that are detrimental and one will blame him/herself for them (Yaghoobei et al., 2014). As a result, he/she uses various justifications to both defend and blame himself/herself for procrastination (Shahbaziyan et al., 2016). On the one hand, one makes excuses for not doing tasks, and on the other hand, he/she waits until the last minute to do those tasks (Khakpoor and Golipoor, 2016). Procrastination may affect health directly and indirectly. The relationship between procrastination and health outcomes may appear surprising, but rushing to complete important tasks at the last minute can be stressful and negatively affect one’s temperament. It may also cause negative self-talk that maintains this negative temperament (Sirois, 2016). In addition, stress and self-care behaviors are interrelated with a combined effect on health (Sirois, 2015).

Emotion and mood may play an important role in treatment adherence. Dialysis and its challenges expose patients to emotional responses, such as loss, anxiety, and depression (Delshad Noughabi et al., 2019). Patients, who control their emotions, perform better in self-care programs, but depressed, anxious, and distressed patients face numerous obstacles to treatment adherence (Yap et al., 2015).

Poor socio-economic status and low level of education are important factors in treatment adherence. High treatment costs and illiteracy associate with treatment non-adherence (Anees et al., 2018). The financial burden of prescribed treatment is an important reason for non-adherence. Furthermore, illiterate patients are unable to recognize and distinguish their prescriptions. They may be unable to comprehend the medication’s dosage instructions as well as the warning labels (Davis et al., 2012).

Treatment adherence is necessary for hemodialysis patients’ health and well-being. To address the negative effects of the aforementioned variables on their physical health, we must first identify the relationship between them and acquire theoretical knowledge and skills. Planning interventional and preventive studies, as well as creating practical knowledge and skills for psychotherapists, doctors and patients can reduce physical and psychological injuries, save costs, and improve physical and mental health of patients. We conducted this study to predict hemodialysis patients’ treatment adherence based on procrastination and difficulty in emotion regulation.

## Materials and methods

### Study design and setting

We conducted this descriptive correlational study in medical centers of Kerman (Shafa hospital, Afzalipur hospital, Samen al-hojaj clinic, and Jawad al-aemeh clinic), Sirjan, and Rafsanjan from July to November 2015.

### Sample size and sampling

We used non-random purposive sampling to select all eligible patients with chronic kidney disease undergoing dialysis. Inclusion criteria included patients aged above 18 years under 3-4-h hemodialysis more than twice a week for more than 3 months, who could walk and eat independently, had consent to cooperate in research, and were able to read and write. Exclusion criteria included patients with cognitive or mental disorders, patients who underwent hemodialysis with previous underlying disease (suicide, drug overdose, and accident), patients with major psychiatric disorders and physical diseases unrelated to dialysis, such as physical disability, cancer, MS and removing the incomplete questionnaires. In regression analysis studies, it has been suggested that more than 100 participants or at least 20 participants for each independent variable should be involved in regression analysis. Green has proposed a method to determine the minimum sample size required in the regression model. He suggested that the minimum sample size should be more than 50 + 8 k, where k represents the number of independent variables. According to Green’s formula, a minimum sample size of 180 people is obtained in this research. But since 180 is the minimum sample size and in order to reduce the measurement error, the researcher increased the sample size. So we selected all eligible hemodialysis patients who referred to medical centers in Kerman, Sirjan, and Rafsanjan from July to November 2015. Finally, we included 218 samples in our study.

### Measurement

The data collection tools in this research were demographic characteristics questionnaire, end-stage renal disease adherence questionnaire, general procrastination scale, decisional procrastination scale and difficulty in emotion regulation scale.

(A) Demographic characteristics questionnaire: This 10-item researcher-conducted questionnaire included sex, age, marital status, the cause of kidney disease, history of hemodialysis, history of peritoneal dialysis, history of kidney transplant, daily and weekly schedule of receiving hemodialysis, history of psychiatric and physical diseases, and insurance.

(B) End-stage renal disease adherence questionnaire: We used self-report questionnaire of Kim et al. (2010) to measure hemodialysis patients’ treatment adherence. This questionnaire measures treatment adherence to hemodialysis attendance, medication use, fluid intake restrictions, and dietary guidelines. The final version of this questionnaire consists of 46 items divided into five sections. The first section examines general information about patients with end-stage renal disease (five items). The final four sections ask about treatment adherence (14 items), medication use (9 items), fluid restrictions (10 items), and diet (8 items). Items 14, 17, 18, 26, 31, and 46 directly measure adherence behaviors, while items 11, 12, 22, 23, 32, 41, and 42 measure patients’ perceptions of the four dimensions of treatment adherence. The responses consist of both Likert scales and yes-no answer format. The adherence behavior subscale is scored by summing the responses to items 14, 17, 18, 26, 31, and 46. The weighting system for scores was based on the importance of that behavior in the clinical outcomes. For example, missing or shortening a hemodialysis treatment has a stronger relationship with patient mortality than other components of adherence behavior, so this behavior gets more weight in the total adherence scores. In addition, the end-stage renal disease adherence questionnaire adjusts scores for 14, 18, and 26 depending on the reasons for not adhering. For example, if patients miss or shorten a hemodialysis treatment due to hemodialysis access problems or physical symptoms, they will receive a full score. The perception/attitude subscale is scored by summing the responses to 11, 12, 22, 23, 32, 41, and 42. The rest of the questions show information about patients’ history of renal replacement therapies. The total score of treatment adherence is the sum of the scores of these five sections. The lowest and highest scores of the questionnaire are 0 and 1,200, respectively, with higher score indicating better treatment adherence. The content validity for each item in four sections was between 0.86 and 1 (0.99 on average). A high content validity score for each item indicates that the items of each section measure the same construct. The test–retest reliability indicated that the correlation scores ranged from 0.83 to 1, so that the self-reported behaviors of adherence and perception were consistent throughout the questionnaire (Kim et al., 2010).

Khalili et al. (2011) was the first one, who used this questionnaire for 30 hemodialysis patients in Isfahan to determine its validity and reliability. A panel of experts confirmed the content validity of the questionnaire. The face validity of this questionnaire was examined to measure hemodialysis patients’ perceptions of the questions. The results indicated an appropriate validity of this questionnaire. Cronbach’s alpha coefficient of 0.75 was calculated for the questionnaire reliability (Khalili et al., 2011).

(C) General procrastination scale: Lay (1986) developed this 20-item scale ranging from one (extremely uncharacteristic) to four (extremely characteristic). He scored negative items (Green et al., 2013; Griva et al., 2013; Magnard et al., 2013; Mellon et al., 2013; Nazari Kamal, 2014; Shahbaziyan et al., 2016; Wang et al., 2017; Mukakarangwa et al., 2018; Tayebi et al., 2019; Asadizaker et al., 2022) reversely. The minimum score is zero, while the maximum score is 80, and the cut-off point is 40. The validity and reliability of the student version are 0.72 and 0.76, respectively. Nazari Kamal (2014) was the first one, who implemented its general version in Iran. To determine the validity of the general version of the questionnaire, Cronbach’s alpha test was performed. First, the questionnaire was used for 30 patients with coronary artery disease and 30 healthy people, and then Cronbach’s alphas were calculated to be 0.78 and 0.81 for patients with coronary artery disease and healthy people, respectively (Nazari Kamal, 2014). The Cronbach’s alpha was 0.64 for the general procrastination scale in this study.

(D) Decisional procrastination scale: Mann developed this 5-item self-report questionnaire to measure procrastination in decisional situations. This scale is graded on a Likert scale ranging from zero (not at all true) to four (completely true), with higher scores indicating a higher level of procrastination. The minimum score is zero, while the maximum score is 20, and the cut-off point is 10. The content validity of this scale is 0.82 and its reliability is 0.64. Hosseini and Khair (2009) investigated its reliability using the internal consistency method and obtained an alpha coefficient of 0.78. They checked the scale validity by factor analysis and principal components. They indicated a general factor in the whole scale that predicted 56.03% of the total variance. The Cronbach’s alpha was 0.76 for the decisional procrastination scale in the current research (Hosseini and Khair, 2009).

(E) Difficulty in emotion regulation scale: Gratz and Roemer, (2001) designed this 33-item self-report questionnaire. The items are scored based on a 5-point Likert scale ranging from zero (almost never) to five (almost always), with higher scores reflecting greater difficulty in emotion regulation. This scale consists of six subscales with high internal consistency (0.93), including non-acceptance of emotional responses, difficulty engaging in goal-directed behavior, impulse control difficulties, lack of emotional awareness, limited access to emotion regulation strategies, and lack of emotional clarity. All subscales have Cronbach’s alphas above 0.80. This scale has a significant correlation with the NMR scale and the acceptance and action questionnaire (Hayes, 1996; Gratz and Roemer, 2001).

Khanzadeh et al. (2012) determined the factor structure and psychometric properties of this scale in Iran. They used this questionnaire for 318 students of Shiraz University. The scale validity for these subscales was between 0.86 and 0.88 using Cronbach’s alpha coefficient and the retest reliability coefficient for these subscales was between 0.79 and 0.91 after 1 week. To increase validity, three items were removed from the questionnaire and the number of questions reduced from 36 in the original version to 33 items (Khanzadeh et al., 2012).

### Data collection

We used questionnaire to collect data in this research. First, we achieved necessary permissions from the Vice-Chancellor for Health and Treatment in Kerman, as well as the hospitals and dialysis centers. Then, we visited all hemodialysis centers in Kerman to conduct the study, including Shafa and Afzalipour hopitals, Samen al-hojaj and Javad al-aemeh clinics. We introduced ourselves and explained the study objective. Then, we gave the questionnaire to eligible patients and assured them that they could withdraw from the study at any time. As patients were unable to sit and fill the questionnaires during dialysis, I read the questions one by one and the patients answered them. The tone and volume of my voice was identical for all patients. Ten patients required more explanations about item 7 of the general procrastination scale and items 2, 6, 4, and 7 of the difficulty in emotion regulation scale. I explained them equally for all patients even those who needed no further explanation. Mr. Khanzadeh checked the validity, reliability, and psychometric properties of the difficulty in emotion regulation scale, so I consulted with him to explain ambiguous questions. I distributed questionnaires to each patient at random and presented the patients with a gift at the end.

Of 297 hemodialysis patients with chronic kidney disease, 180 patients participated in the study that were from Kerman and all the neighboring cities, districts and villages due to the lack of access to dialysis facilities. To increase the sample size and reduce the sampling error, we selected Sirjan and Rafsanjan that had the most cultural, social and economic similarities with Kerman, so the sample size increased to 230 individuals. We removed 12 incomplete questionnaires, and then analyzed 218 questionnaires. As patients from Bam, Jiroft, Kahnuj, Baft, Rayen, Bardsir, Zarand traveled to Kerman to visit nephrologists for more advanced tests, a number of them participated in the research and completed the questionnaire.

### Data analysis

We used SPSS18 to analyze the data. Then, we coded them after gathering the questionnaires. We used descriptive (mean and standard deviation) and inferential (Pearson correlation coefficient and multivariate regression analysis) statistics to analyze data. A significance level of 0.05 was considered.

### Ethical considerations

We started this study after receiving permission from Kerman University of Medical Sciences, the officials of hemodialysis centers, and patients’ consent. We assured the research units that their participation was voluntary and would have no effect on the healthcare provision. We also gave necessary information and explanations about the research and items of the questionnaire.

## Results

The study results suggested that the mean age of the samples was 54.11 ± 14.78 years. The majority of them were male and married. Only 13.8 percent of them were employed. Most of them had an education level lower than diploma, with poor to moderate income. The majority of them were citizens and had personal houses. Most of them had moderate access to utilities. Our results found a significant relationship only between employment status and treatment adherence (*p* = 0.003; Table 1).

**Table 1:** The relationship between demographic characteristics and treatment adherence.

Variable	Frequency (%)	Percent	Treatment adherence		Statistical test	*p*-value
			Mean	SD		
Sex						
Female	86	39.6	856.32	153.74	*T* = 1.07	0.28
Male	131	60.4	833.12	157.98		
Marital status						
Married	162	74.3	843.51	151.58		
Single/divorced/widowed	56	25.7	839.29	170.75	*T* = −0.17	0.86
Employment						
Unemployed	42	19.2	783.93	183.45	*F* = 4.81	0.003
Employed	30	13.8	791.67	162.99		
Housewife	61	28	876.64	127.87		
Retired	85	39	864.94	147.84		
Education level						
Middle/high school	143	66.8	849.51	156.72	*F* = 0.69	0.5
Diploma	45	21	824.44	147.66		
Academic	26	12.2	820.19	174.36		
Income						
Poor	94	43.3	845.97	147.36	*F* = 0.35	0.71
Moderate	88	40.6	834.72	165.04		
Good	35	16.1	860	155.68		
Residential place						
Urban	156	71.6	842.13	155.89		
Rural/suburban	62	28.4	843.15	158.77	*T* = −0.04	0.97
Housing						
Personal	180	82.6	842.63	155.22	*T* = 0.04	0.97
Others	38	17.4	841.45	163.71		
Access to utilities						
Poor	69	32.3	843.06	159.29	*F* = 0.46	0.63
Moderate	79	36.9	853.54	154.68		
Good	66	30.8	828.41	156.4		

Table 2 indicated that diabetes was the cause of kidney disease in most of the patients. The majority of them had no history of peritoneal dialysis or kidney transplant and they were receiving hemodialysis between 1 and 4 years. The cause of kidney disease had a significant relationship with treatment adherence (*p* = 0.02).

**Table 2:** Description of the mean and standard deviation of treatment adherence, procrastination, and difficulty in emotion regulation.

Variable	Mean	SD	Minimum	Maximum
Dialysis treatment adherence	842.42	156.352	300	1,200
Medication use	164.68	51.611	0	200
Dietary guidelines	66.28	64.21	0	200
Fluid intake restrictions	75.69	72.03	0	200
Attendance	541.73	101.59	200	600
General procrastination	28.68	10.613	3	59
Decisional procrastination	6.70	4.812	0	20
Difficulty in emotion regulation	57.69	15.661	15	100
Non-acceptance of emotional responses	10.68	8.541	0	28
Difficulty engaging in goal-directed behaviors	10.39	2.612	0	18
Impulse control difficulties	11.88	2.170	4	19
Lack of emotional awareness	14.55	3.874	2	21
Lack of emotional clarity	6.90	6.440	0	24
Limited access to emotion regulation strategies	4.10	3.475	0	12

Our results demonstrated that the mean score of treatment adherence was 842.42 ± 156.352 and the scores ranged from 300 to 1,200. The higher the mean adherence score, the higher the adherence rate. Attendance had the highest average indicating that patients adhered to attendance more than other dimensions, while adherence to dietary regimen had the lowest average. The scores for the general procrastination scale ranged from 3 to 59, with a mean of 28.68 and the standard deviation of 10.61. Decisional procrastination scores ranged from 0 to 20 with a mean of 6.70 and a standard deviation of 4.81, indicating low procrastination. The total mean score of difficulty in emotion regulation was 57.69 with a standard deviation of 15.69. Among the subscales of difficulty in emotion regulation, lack of emotional awareness had the highest average and limited access to emotion regulation strategies had the lowest average. The mean score of the patient’s perception was 68.43, with a standard deviation of 10.54, and the distribution of scores was between 11 and 68, indicating that patients had a negative perception of their illness and treatment. The patient’s perception of the doctor’s empathy showed a mean of 20.19 and a standard deviation of 13.36. The pre-treatment awareness scores were between 0 and 16, with a mean of 2.78 and the standard deviation of 3.86. Both the patient’s perception of the doctor’s empathy and awareness were weak (Table 3).

**Table 3:** The relationship between treatment adherence, general procrastination, decisional procrastination, and difficulty in emotion regulation.

Variable	Pearson correlation coefficient		
	General procrastination	Decisional procrastination	Difficulty in emotion regulation
Medication use	−0.22 **	−0.16*	0.02
Dietary guideline	0.08	−0.1	0.003
Fluid intake restrictions	0.11	0.08	0.02
Attendance	−0.14*	0.04	−0.1
Total	−0.06	−0.028	−0.03

*p < 0.05, **p < 0.01.

Treatment adherence had no significant relationship with the variables of general procrastination, decisional procrastination, and difficulty in emotion regulation (*p* > 0.05). Among the dimensions of treatment adherence, medication use had a significant, weak, and inverse relationship with general and decisional procrastination. In addition, treatment adherence had a significant, weak, and inverse relationship with attendance and general procrastination (*p* < 0.05 and *p* < 0.01) (Table 4).

**Table 4:** The relationship between clinical characteristics and treatment adherence.

Variable	Frequency	Percent	Treatment adherence		Statistical test	*P*-value
			Mean	SD		
Cause of kidney disease						
Diabetes	98	45.2	865	143.56	*F* = 3.86	0.02
Hypertension	48	22.1	790.63	154.85		
Other diseases	71	32.7	850.35	165.64		
History of peritoneal dialysis						
Yes	12	5.5	829.17	188.85	*T* = 0.28	0.78
No	205	94.5	842.18	154.45		
History of kidney transplant						
Yes	27	12.4	816.67	177.59	*T* = 0.91	0.36
No	191	87.6	846.08	153.27		
History of hemodialysis						
3–6 months	31	14.3	813.87	155.37	*F* = 0.86	0.49
7–12 months	38	17.6	824.34	132.99		
1–2 years	54	25	837.04	170.72		
2–4 years	55	25.5	865.45	136.97		
More than 4 years	38	17.6	863.16	156.16		

The bivariate analysis reported a significant relationship between age and treatment adherence (*r* = 0.21 and *p* = 0.002). In addition, we found a significant difference in the treatment adherence score based on the employment status and the cause of kidney disease (Tables 1, 2). To determine predictors of hemodialysis patients’ treatment adherence, we included the variables with *p* < 0.2 (age, employment status, and cause of kidney disease) into the stepwise multivariate linear regression model. Our results showed a relationship between age, the cause of kidney disease, and treatment adherence. In other words, the older the age, the higher the treatment adherence, and patients whose cause of kidney disease was hypertension had less treatment adherence than other patients (Table 5).

**Table 5:** The predictors of treatment adherence.

Model	Unstandardized coefficients		Standardized coefficients	*t*	*p* value	95.0% Confidence interval for B	
	B	Std. Error	Beta			Lower bound	Upper bound
Constant	756.22	41.48		18.23	<0.001	674.45	837.98
Age	1.85	0.72	0.17	2.58	0.01	0.44	3.26
Cause (hypertension)	−59.09	25.4	−0.16	−2.33	0.02	−109.16	−9.02

*F* = 7.3, *p* = 0.001, Adjusted *R*^2^ = 0.06.

## Discussion

We aimed to study the relationship between hemodialysis patients’ treatment adherence, procrastination, and difficulty in emotion regulation. Our results indicated that among the dimensions of treatment adherence, medication use had a significant, weak, and inverse relationship with general and decisional procrastination. We also found a significant, weak, and inverse relationship between attendance and general procrastination. But there is no significant relationship between treatment adherence, general procrastination, and decisional procrastination. In justifying these results, it can be stated that from a psychological point of view, to create change, one should focus on specific behaviors, not on general adherence. Therefore, in order to improve and promote adherence to treatment in hemodialysis patients, it is possible to focus on specific areas such as medication and attendance. So that better and more useful results can be achieved in the field of adherence to treatment in patients. We were unable to find any research on the relationship between procrastination and treatment adherence in hemodialysis patients or other patients, so we were unable to compare our findings to those of other researchers. However, procrastination is a psychological trait (Sharifi Rahnemo et al., 2021), so we reviewed similar articles. Allahmoradi et al. (2022) showed that depression and personality components (neuroticism, introversion, flexibility, agreement, responsibility) predict adherence to treatment in addicted patients, but among these variables, the neuroticism component is stronger. Also, the components of personality and adherence to treatment are predictors of anxiety, among which the predictive flexibility is stronger. Finally, the components of personality and adherence to treatment, except for responsibility, are predictors of stress, among which the predictive flexibility is stronger (Allahmoradi et al., 2022). In another study, Wessels-Bakker et al., (2022) found that symptoms of anxiety and medication adherence were significantly and positively related in transplant recipients. They found no association between depressive or post-traumatic stress symptoms, and medication adherence (Wessels-Bakker et al., 2022). Omranifard et al. (2017) showed that there was no significant relationship between anxiety and compliance of kidney transplant patients. The reason may be that as the duration of the disease increases, the severity of the disease’s anxiety will decrease and due to familiarity with the disease and its complications, their fear and anxiety will also decrease. At the same time, based on the functional model, moderate anxiety may even lead to increased therapeutic cooperation.

Procrastination is common in industrialized societies due to a lack of time and planning. We studied procrastination in a health sector in a developing country and found that self-care behaviors were less important in developing societies and procrastination in the health sector was common. People are still unaware that procrastinating on health-related behaviors can result in irreversible damage. Our results indicated no significant relationship between treatment adherence and difficulty in emotion regulation because hemodialysis patients were anxious and depressed due to the disease severity. Therefore, it does not capture the emotional regulation issues in dialysis patients. Although we controlled psychological diseases by checking psychiatric drugs, more control is necessary with the help of tools that measure anxiety and depression. In addition, lack of significant relationship may be due to confounding variables. Studies suggested that anxiety, depression, and alexithymia were very common among hemodialysis patients (Ahmadzadeh and Mehdi, 2012; Mirbagher-Ajorpaz et al., 2016), so we assumed that most of these patients had difficulty in emotion regulation that could affect their treatment adherence. Wierenga (2017) found no significant relationship between emotion regulation and general adherence in women with heart failure. However, Abdoli et al. (2021) reported a direct relationship between emotion regulation and treatment adherence in women with breast cancer undergoing chemotherapy. Their results were contrary to our results due to different statistical population, sample size, research purpose, and data collection tools.

We found limited studies on the relationship between emotion regulation and treatment adherence, so we were unable to confirm the relationship between them. As emotion regulation strategies associated with treatment adherence, we concluded that emotion regulation and health were interrelated. Applying emotion regulation skills in the face of stressful situations reduces negative emotions and stress, as well as physiological stress responses, including endocrine and autoimmune responses that improve the mental and physical condition. Therefore, emotion regulation skills play an important role in preventive interventions and psychological treatments for a variety of physical conditions, psychological diseases and disorders (Palmer and Alfano, 2017). Emotion avoidance can have adverse consequences, such as disease progression and acceptance. Expressing feelings may increase physical and mental health. Unresolved emotions and mind rumination about negative emotions can negatively affect the patient’s health. For example, these emotions cause a chronic increase in the activity of the sympathetic system. In addition, emotion inhibition can delay help-seeking behaviors because it prevents patients from detecting symptoms, so they fail to perform health maintenance behaviors and adhere to treatment (Brockman et al., 2017). Debilitating and threatening complications affect all physical, mental, and social aspects of patients’ lives and pave the way for negative emotions, such as anxiety and depression. On the other hand, emotional disorders can accelerate the disease progress and have a negative effect on the disease management (Naragon-Gainey et al., 2017).

The regression analysis showed a relationship between age, the cause of kidney failure, and treatment adherence. In other words, the older the age, the higher the treatment adherence, and patients whose cause of kidney failure was hypertension had less treatment adherence than other patients. Some studies supported our study regarding the relationship between age and treatment adherence. Sheikh et al. (2022), Varghese (2021), Rafiee-Vardanjani et al. (2013), and Khalili et al. (2011) found a significant relationship between hemodialysis patients’ age and treatment adherence, so that older patients were more adherent than younger patients. They also found an association between income, sex, education, marital status, and treatment adherence that was not consistent with our results. Rafiee and Shafie (2017) reported no significant relationship between age, sex, marital status, level of education, income, the cause of kidney disease, and treatment adherence. Ross (2017) and Alhomayani et al. (2021) associated marital status with treatment adherence, so that single, widowed, or divorced patients were more at risk of treatment non-adherence than married patients were. They also found that age, race, cause of end stage renal disease, sex, and quality of hemodialysis had no association with hemodialysis adherence (Ross, 2017; Alhomayani et al., 2021). Their results also contradicted our results, possibly due to differences in patient age, sample size, and data collection tools. In addition, target populations were different that might have influenced the results. Different results suggest the influence of many factors on patient treatment adherence. Therefore, other studies should address specific geographical areas with different cultures.

This study also had limitations. We conducted this study on patients in Kerman province that limited generalizing the findings. We ignored psychological factors, such as anxiety and depression, and the difficulty in emotion regulation scale was not proportional to patients’ literacy. Data collection was in the form of self-report, which can be a major limitation. Because the authors only rely on their report. Also, the collection method prevents the anonymity of the participants, and as a result, patients may not give accurate and correct answers to the questions.

We recommend our study on other patients in different cultures. Although the problem is a physical disease, cultural factors can affect the cognition, belief, and level of awareness of patients. Other studies should examine the mediating role of psychological variables, such as self-efficacy, patients’ attitudes toward the disease, procrastination and treatment adherence of hemodialysis patients as well as other samples.

We recommend that future studies address the role of clinical psychological variables, such as anxiety and depression, compare psychological status of these patients with those who are not in the end-stage renal disease, investigate the emotional state of patients using other tools, and validate the procrastination scale for measuring procrastination in health and self-care behaviors.

## Conclusion

Our results showed that medication use and attendance are related to procrastination in hemodialysis patients. Also our results suggested that age and cause of kidney disease were the predictors of treatment adherence in hemodialysis patients. Patients’ compliance with treatment instructions is very important, so that their treatment non-adherence causes problems and complications, and disrupts the course of treatment. Therefore, it is necessary to identify the factors influencing non-adherence of patients undergoing hemodialysis and improve their treatment adherence, and thus their quality of life. Anyway, we need more studies to confirm our results.

## Data availability statement

The original contributions presented in the study are included in the article/supplementary material, further inquiries can be directed to the corresponding authors.

## Ethics statement

Ethical approval was not provided for this study on human participants because this study was conducted at a time when the university rules were such that there was no need to obtain a code of ethics to conduct research, and we could conduct the research only based on the approval of the relevant university. The patients/participants provided their written informed consent to participate in this study.

## Author contributions

FB collected the study data. FB and ZD wrote the manuscript. All authors read the article and made the necessary checks for its correction, approved the article.

## Conflict of interest

The authors declare that the research was conducted in the absence of any commercial or financial relationships that could be construed as a potential conflict of interest.

## Publisher’s note

All claims expressed in this article are solely those of the authors and do not necessarily represent those of their affiliated organizations, or those of the publisher, the editors and the reviewers. Any product that may be evaluated in this article, or claim that may be made by its manufacturer, is not guaranteed or endorsed by the publisher.
